# Benefits and challenges perceived by patients with cancer when offered a nurse navigator

**DOI:** 10.5334/ijic.629

**Published:** 2011-10-07

**Authors:** Marianne K Thygesen, Birthe D Pedersen, Jakob Kragstrup, Lis Wagner, Ole Mogensen

**Affiliations:** Department of Gynecology and Obstetrics, Odense University Hospital, and Institute of Clinical Research and Research Unit for General Practice, Faculty of Health Sciences, University of Southern Denmark, Denmark; Research Unit of Nursing, Institute of Clinical Research, Faculty of Health Sciences, University of Southern Denmark, Denmark; Research Unit for General Practice, Faculty of Health Sciences, University of Southern Denmark, Denmark; Research Unit of Nursing, Institute of Clinical Research, Faculty of Health Sciences, University of Southern Denmark, Denmark; Department of Gynecology and Obstetrics, Odense University Hospital, Sdr. Boulevard 29 5000 Odense C, Denmark, and Institute of Clinical Research, Faculty of Health Sciences, University of Southern Denmark, Denmark

**Keywords:** integrated care, nurse navigator, continuity, cancer, patients view, qualitative study

## Abstract

**Introduction:**

Lack of communication, care and respect from healthcare professionals can be challenges for patients in trajectories of cancer, possibly accompanied by experienced fragmentation of the care, anxiety and worries. One way to try to improve delivery of care is additional help from nurse navigators (NN) offered in a predefined shorter or longer period, but patients’ experiences with this have seldom been investigated.

**Aims:**

To explore experiences of nurse navigation offered in a short period of a longer subsequent part of cancer trajectories by patients who can use the help on offer.

**Methods:**

The NNs worked from one hospital department with patients in the transition between primary care and a university hospital before admission. A phenomenological-hermeneutical longitudinal study was performed from referral and until two months after discharge from the hospital. Semi-structured interviews with five patients who could use the help from an NN provided data for the analysis, which started open-minded.

**Results:**

Affectional bonds were made to the NN and patients felt that they benefited from her presence and her help, which they requested until one month after discharge. They were disappointed and felt rejected when the contact to the NN stopped.

**Conclusion:**

In efforts to increase quality of care for patients with cancer we recommend an increased awareness of cultural areas within the healthcare system, which may be an impediment to good communication. Moreover, we recommend paying special attention to critical periods in cancer patients’ trajectories, as well as to the theory of attachment to supplement thoughts of continuity of care and coordination in the care for women. In short, it is fine to offer additional help to those who can use it, but in practice as well as in research we recommend awareness of how and when to stop the help, to prevent patients from feeling hurt.

## Introduction

Patients with cancer do not receive the help they could wish for [1–3]. According to national surveys from Denmark and the UK lack of communication, care and respect are experienced in relation to healthcare professionals [1, 2]. Uncertainty [4], worries and fear [5], experienced fragmentation [6–9] and transitional problems in the healthcare system [5, 10] can present challenges in the trajectory as well. Use of nurse navigators as additional persons in the healthcare system is an emerging trend expected to tackle such problems [11–16], but evidence-based knowledge on patients’ experiences over time with such help is limited.

Professional navigators help cancer patients “not only travel the healthcare maze in a more timely fashion, but their psychosocial well-being and quality of life may also be enhanced” [29, p. 17]. Nurse navigators (NN), where nurses do the job of professional navigation, are now to be found in the healthcare system in the US, Canada, and Australia, and are recommended based on thoughts of continuity of care as well as supportive care [11]. Continuity of care embraces communication, management of care, and the relation to others, which patients should experience as coherent, connected, and consistent with their medical needs and their context [18]. This leaves the NN with several key elements of work in relation to the same patient, in the areas of assessment, education, coordination, and support as illustrated in Box 1 [11]. Whether the NNs are rooted in one single hospital department or in centers, their work should not duplicate or overlap the work of others [12, 14, 17].

There are many supplementary roles to the healthcare system aiming to help the patient co-ordinate care [19]. All are described as frames and mostly filled out with exact work in the specific situations. An NN’s role is similar to other roles, and may be complementary to case management. The case management role is not clearly defined, but has been used in nursing care in the US for decades in order to decrease costs as well as to help patients through a part of a disease trajectory. The role has developed over time and can now be divided into three generations [20], where important features of third generation case management, as well as for the NN role, are the holistic approach to patients and a focus on empowerment [11, 20]. Moreover, the NN have a focus on availability to patients [11–16]. To our knowledge only one study [12] has qualitatively investigated cancer patients’ experiences with help from an NN in a longitudinal fashion. In this study by Fillion et al. both male and female patients were offered help from an NN from diagnosis and until the end of the care trajectory, and they report on patients’ experiences of reduced anxiety and increased empowerment. Furthermore, authors who have investigated patients’ experiences of longer duration of help from NNs report the NN to be a helping resource who reassure and give tailored information [11–13, 16]. Shorter periods of help are offered too. Not all patients are able to take advantage of help from an NN, regardless of whether it is offered in a short (unpublished work) or a longer period [12, 16]. The aim of this paper is to explore experiences of nurse navigation offered in a short period of a longer subsequent part of cancer trajectories by patients who can use the help on offer.

## Method

A qualitative longitudinal study with a phenomenological-hermeneutical approach was conducted among patients who accepted to participate before meeting an NN. This paper reports from a part of a larger study aiming to investigate how women with cancer experience an offer of nurse navigation in the diagnostic period and while waiting for primary treatment. Those who could use the help from an NN provide data for this paper.

### Nursing contact and physician contact in outpatient and hospital setting

The NNs were from a gynecological department at a university hospital in the Region of Southern Denmark that receives patients with suspected or diagnosed gynecological cancer from the entire Region of Southern Denmark (1.2 million inhabitants). The NNs worked in and from the outpatient setting, localized in one end of the in-hospital ward. Help from one NN was offered to a patient from the day after the referral had reached the outpatient clinic, and until the patient was admitted to the ward for planned surgical removal of (possible) cancer. After admission the patients had no further formal contact with their specific NN, but were instead appointed a nurse from the ward as contact person. At discharge the patients were offered to call the nursing station in the ward from which they were discharged, if some questions arose in the first days after coming home. The patients could have contacts with a new physician, nurse (or radiotherapist) each time they were in contact with the healthcare system in their trajectory of cancer, including several healthcare professionals during the in-hospital period, but they had only one NN.

### Nurse navigation

The role of the NN was designed by clinical staff (nurses and a physician) without reading specific literature on nurse navigation, and reflected what they thought feasible and felt could help the patients. The NNs were female and they had no earlier experiences with NN work. Their competences were made clear prior to this study; they had more than five years of experience in care for gynecological cancer patients, and in-depth knowledge of the hospital trajectory of cancer from a professional view. They had excellent communication skills and had knowledge of information commonly given to patients with gynecological cancer, from suspicion being aroused and to discharge, and some knowledge of information given at follow-up post surgery. Furthermore, they were able to manage the booking system and the electronic patient journal of the hospital, and they had problem-solving skills, like overview as well as ability to react. Moreover, they had knowledge about support opportunities, for instance patient organizations and the Danish Cancer Society. The NN collaborated with healthcare professionals as well as others outside the outpatient clinic, when needed. She reached out to help patients who lived at home and were examined in other units of the hospital, other hospitals or at private physicians. Patients came from different municipalities with differences in availability to systems for delivery of help to e.g. transportation. An NN was proactive in the first contact to the patient by calling, if the patient allowed it. The NN had the time needed, and in a supportive talk they investigated if the NN should help immediately by, for instance, empowering with information or education not available in pamphlets, rebooking scheduled appointments, jointly making plan of action complementary to a plan of investigations, or linking to other resources if others could help. The NN was present at the outpatient clinic and worked as the ambulatory nurse. She followed the patient and her relative to the appointment with the physician, repeated the information afterwards in a more tailored fashion, answered questions and they jointly created a short-term plan of action in relation to the plan of treatment. The NN was always on stand-by at the telephone during office hours until admission, and could be helpful with coordination, information and counselling as well as a supportive talk, until admission or referral further on. The patients were informed twice of this restricted period of availability, as well as of the help offered; by first author, when they were first offered a nurse navigator to contact them, and again by the specific nurse navigator, when she first called them.

### Participants

We contacted consecutively by phone 14 patients referred to the ward, who later underwent surgery for cancer. The purpose of the study and the participants’ contribution were explained, and they were asked to consider participation. We included 11 patients the day after referral had reached the outpatient clinic and among those five could use the help from the NN. These five presented diversity in age [median age 54 (range 37–76 years of age)] and in socio-demographic characteristics as well as illness- and disease-specific characteristics (Table 1) with, for instance, different diagnoses primarily found at different stages, radical or extensive surgery, and regarded cured after surgery or not. Three declined to participate [median age 53 (range 51–65 years of age)] because they felt lack of energy or would not use time on it. This article focuses on the five women, who could use the help from the NN.

### Data

Techniques to let participants’ expressions come forward were found appropriate to use, as the purpose was to examine patients’ experiences. A shared background with the participant before semi-structured interviews was desirable, and several techniques were used. Diaries can provide insight into experiences of others as they unfold in writing, close to the time of experience [21, 22], and a semi-structured diary was sent by mail to all included, together with an information sheet on the study and contact information to the NN. As coherent written essays including negative emotions are considered to be rewarding in stressful situations [23], and as the reward of writing could affect the participants’ use of help from the NN, only a quarter of an A4 sheet was left in the diaries for a coherent narrative for each contact with a healthcare person. The diaries were kept until two months after discharge to hold on to experiences of help from healthcare professionals as well as to hold on to feelings and emotions. They were kept by all participants but one, who only filled in a minor part. Moreover, observational studies can provide a shared experience [24]. The outpatient visit was the only time in the trajectory the NN and the participants met each other face to face, and observational studies at this visit were conducted if the participants allowed it. Observations were conducted following all five participants. Together with the diaries the observational studies provided a shared background and were used entirely to qualify first author to ask questions of relevance in the interviews. Narratives obtained through semi-structured interviews are a good way of gaining insight into the world experienced by the narrator [25]. Interviews were held at time of discharge (no. 1) to hold on to experiences, and again two months after discharge (no. 2) to look back on the trajectory with a distance from the acute period, but not so great a distance that experiences with the NN had been forgotten. All interviews were conducted at a place chosen by the participant, and if this choice was not their home, a room was provided at the hospital to ensure they could talk in private. The first author conducted all interviews with an open, interested attitude, focusing on getting the participant to narrate. Several techniques were used to guide and support participants: a semi-structured interview guide including themes and suggestions to open-ended questions [25] like “How have you made use of healthcare professionals?” (e.g. the nurse navigator) and “What was your experience when it was suspected that you had cancer?”; and an elicitation technique which was specially developed in the study [26]. It helped participants to remember and talk about parts in the trajectory, while the participant drew graphs of emotions. All 10 interviews were recorded, lasted on average one hour, and were transcribed verbatim. The 10 interviews provided the data for further analysis.

### Analysis

A phenomenological-hermeneutical approach to analysis and interpretation of participants’ experiences was considered appropriate, and a method inspired by Ricoeur’s theory of interpretation [27] was followed. Interpretative methods have in the Nordic countries been inspired by this theory since the 1990s [28, 29]. It was carried out by the first author in three levels. A first level labeled ‘naive reading’, where the text was read several times to grasp its meaning as a hole. A second level where a structural analysis was performed in a sequential process running in a spiral fashion between understanding (what is talked about) and explanation (what is said) and ended up with themes and subthemes in the text. Work in these two levels was continued until the structural analysis validated a naive reading where no contradictions were accepted. A third level comprise a comprehensive understanding and a discussion where the results were related to relevant theory and other studies. First author made the text-faithful analysis, and critical discussions about results were made with co-authors along the process of interpretation.

### Ethics

Participants were verbally informed about the study by phone and again face-to-face in the waiting room at the outpatient clinic, where informed consent was signed. No names are used in the following in order to maintain anonymity. The study adheres to the Declaration of Helsinki [30] and Ethical Guidelines for Nursing Research in the Nordic Countries [31]. The Biomedical Research Committee System Act at the Scientific Committee for Southern Region in Denmark does not apply to this project. The Danish Data Protection Agency gave formal consent to the study.

## Results

In the naive reading and structural analysis two distinct themes were found to describe the experiences of nurse navigation: benefits and challenges. Benefits comprised a sub-theme: help and mutual connection (Table 2), and challenges were divided into the sub-themes: ‘Break of mutual connection’ and ‘Lack of help’ (Table 3). In the following we will elaborate on the central findings from the structural analysis (Tables 2 and 3), which contain quotations from both first and second interview. From first to second interview (no. 1 and 2) the essence of the narrative with regard to the aim of this article did not change. All quotations are chosen where a participant best explained the situation as she felt it.

### Benefit: help and mutual connection

All participants experienced a mutual connection with the NN, which was special for this healthcare person (Table 2, lines A, B, C). They had quickly built up confidence in the NN, for some explained by the NN’s knowledge of their trajectory so far, and her ability to provide them with a picture of what to expect in the near feature. The NN was experienced as a trustworthy and forthcoming person who offered her attention and could and would help them over time. This was of great value to them, as they found themselves being particularly vulnerable and happy to have the same point of contact. Participants felt reassured to know they could call her, although not all used this opportunity. Some contacted her throughout the available period for further information and counseling and regarding problems with coordination, and some only talked to NN twice; (a) at the time NN contacted them, and (b) at the outpatient clinic. These participants collaboratively used help from an NN in all categories offered: coordination, information and counseling as well as supportive talk. Participants described themselves as being in a particularly difficult situation where information was hard to incorporate. They appreciated getting information they could understand, including explanation of physicians’ information and the importance of repeated examinations as well as what to expect in the near feature. When participants had difficulties in getting an overview they were happy to receive counseling and help from the NN, and they jointly made plans of action complementary to plans of investigations or plans of treatment. Sometimes the NN participated in the plans, which the participants were very pleased about. This could, for instance, be by making different providers and the municipality cooperates on the same plan of action, or by guiding patients around the University Hospital for their diagnostic investigation appointments. The NN was valued for her time given and her action taken to immediately help them. Moreover, they valued her ability to reassure and strengthen their resources to deal with difficult tasks, like telling the children that their mum has cancer, or asking an ex-husband to look after the children in an acute period. In this way the participants felt they received help to adjust parts of their lives to a situation with acute cancer. In a period where the participants primarily preferred to think of anything but the cancer, the NN became someone special—she was ‘perfectly nice’.

### Challenge: break of mutual connection

Most patients had pain and had to walk the corridors several times a day as part of a regime after surgery, and some had a good contact to nurses on the ward, others had not. Some healthcare professionals did not greet them when they passed each other in the corridors of the hospital ward, but participants commented especially on situations where the NN did not greet them (Table 3, line A). The NN’s attitude was far from what was expected by these participants and they felt rejected and a disappointment and indignation in relation to this. Whether or not the participants felt healthcare professionals to be very big authorities and in this way did not wish to provoke the system, some of those who did not feel rejected in the corridors, contacted the NN after discharge for further help. The municipality did not offer help with rehabilitation, and the NN was requested to help with an easily available and progressive rehabilitation. Also these participants felt rejected and disappointed, now because the NN immediately linked to other resources instead of helping herself. The NN became a disappointing healthcare professional who by rejection gave them extra challenges in their trajectory.

### Challenge: lack of help from the NN

The limited period of help from the NN as well as the possibility of calling the nurses on the ward and their general practitioner was known by all participants. In this context a request for help from the NN after discharge was only put forth when participants did not feel rejected by her in the in-hospital setting (Table 3, line B). Only those who felt rejection by the NN called the ward after the discharge. A request to be included in the scope of the NN until at least one month after discharge was put forward. All had fear of cancer (and death) and felt a special vulnerability in the period from referral and until one month after discharge. In the period right after discharge the physical problems were in focus. Fading postoperative physical symptoms left room for increased thoughts on both the partly repressed situation with fear of cancer, and the period they have passed, as well as how to manage from now on. In this field the NN was specifically preferred as the one to talk to in supplement to close relations, and rather than with a physician, as the participants were in need of tailored information in a period where they felt insecure and vulnerable, and the physician could be too big an authority. It was expected that the NN could decide whether what they experienced was normal or not in relation to the trajectory so far, and moreover to outline the near future. In this way the NN was requested as a nurse with special skills in communication and special knowledge in these kinds of trajectories at both the general and individual level.

## Comprehensive understanding and discussion

In this study benefits of an available nurse navigator (NN) were clearly experienced by the participants, but so were challenges, because the participants did not get the contact to the NN and the help from the NN they insisted on. The insistence on contact with the NN expressed by participants through most of the followed period could be explained by one of the ideas of continuity of care [18], with personal contact to one person being very important. In this regard the NNs created contact with cooperating healthcare professionals as well as providers, municipalities, patient organizations, other patients and relatives. However, the only contact further on was provided to the physician in the outpatient clinic, who was not always the physician who primarily followed the patient further on. Within the thoughts of Integrated Care [8] this is not optimal as the continuity created by one or few persons is desirable. Those who created the NN role seem not to have focused on the matching of expectations between patient and providers in a wider sense. However, thoughts of continuity of care cannot explain the very special position the NN obtained in the course of illness, and why the distant attitude of the NN provoked such an emotional reaction. With continuity as an essential part of a theory descending from development psychology, Bowlby’s theory of attachment offers an explanation developed on empirical data on children, but reformulated with regard to adults [32], and found to be universal [32–34]. When an adult is sick or scared, an inborn tendency is activated to seek attachment to a clearly defined individual who is counted on to be able to do better in the world, a person who is counted on to be stronger and/or wiser. Attachment is to be understood as a person being “strongly disposed to seek proximity to and contact with that individual and to do so especially in certain specified conditions ... [and] attachment behavior ... refers to any of the various forms of behavior that the person engages in from time to time to obtain and/or maintain a desired proximity” [33, p. 28]. If the chosen person accepts being an available and helpful caring person the emotional (or affectional) bonds that are made have a protective function, and a secure base is under construction. The seeking of attachment is like that a child is seeking to its mother, and this behavior has a protective function. Emotional bonds will take several years to establish [33]. However, as seeking of attachment is an inborn tendency a healthcare professional in a part of a person’s trajectory of cancer can be chosen as the potentially ideal attachment figure, even though a reasonable part in the person knows of a limited period of help. The NN offered herself as an attachment figure and was chosen as such by the participant. The NN became someone special due to the proximity and the seeds of affectional bonds the participant felt were created in the mutual connection. The start and end of such relationships can be very emotional [34], and in this way the proximity and seeds of affectional bonds felt created extra vulnerability among these participants when they later met a non-concerning attitude from the NN. The participants felt rejected and disappointed, and this turned into extra challenges for them; they lacked help and had to overcome the disappointment in an especially vulnerable period. According to Bowlby, individuals can explore much by themselves and be away from their secure base for longer periods, if they are confident in getting help from him/her, exactly when needed. This demands a special behavior from both parts [33, 34]. From the NN the demand was to bring signals of being the selected attachement figure to the participant, which included greeting them when passing each other in the corridors, and not link to other resources, when the help could be given by the NN.

Healthcare professionals’ role as attachment figures to patients is not a totally new idea. In 2002 Grieve et al. recommended seeking of attachment as a coping strategy patients can use on the surgical ward [35]. Moreover, recent research has shown that general practitioners are given such a role, which might explain why it can be difficult to change general practitioner [36]. The participants in this study did use different kinds of help and different amounts of help from the NN. Others found the person’s attachment style in a complex way being of importance for the extent of attachment [32]. Our data do not allow for such analysis, but could be the focus for further research. However, everybody seeks attachement to some extent [32, 36]. As a person who is counted on to be able to do better in the world of healthcare and sickness, a healthcare person is a potential attachment figure.

**Figure 1.**
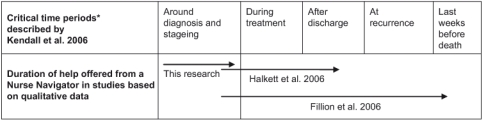
Duration of help offered by nurse navigators in different studies—marked by arrows—in relation to *critical time periods in cancer trajectories identified by cancer patients and their carers in the UK.

Fear of cancer (and death) and a special vulnerability were found among the participants in up to one month after discharge. This corresponds to several of more critical time periods identified in a qualitative study by Kendall et al. who took the patients’ and carers’ view (Figure 1) [37].

The critical periods found by Kendall et al. represent periods where we must assume cancer patients to be more vulnerable, probably feeling more sick and/or scared. The additional help offered to the participants we followed covered (a part of) the first critical period; around diagnosis and staging, and for all it stopped right before a new critical period: during treatment (Figure 1). Our results show that patients were disappointed to lose the help from the NN. This corresponds with results from a study with similar short period of help from an NN offered to male and female patients with non-small cell lung cancer before treatment at a cancer center in the US [15]. Our participants requested help at least minimum one month after discharge, but it is notable that we did not follow them during a period of recurrence. In the study by Kendall et al. cancer patients and their carer wished for available help from primary care, from the diagnostic phase and until the end of the care trajectory, apart from periods with recovery [37]. This nearly corresponds to the period of help offered from an NN to both male and female patients with head and neck cancer in a qualitative longitudinal study by Fillion et al. in Canada [12]. From diagnosis to the end of the care trajectory, cancer patients (and their carers) were offered the possibility of contact and additional help from an NN (Figure 1). Moreover, Halkett et al. [16] have investigated Australian breast cancer patients’ experiences with help from an NN from diagnosis through treatment and follow-up (Figure 1). The patients (and carers of patients with head and neck cancer) experienced values found in these two studies were in part like our results; the helping relationship, tailored information, the availability of the NN, and the time or awareness granted, as well as empowerment and reassurance or reduction of anxiety. Two studies using questionnaire or distress scale report on such kind of benefits as well [11, 13]. Moreover, we would add the power to make things happen, to create an overview and being the single point of contact. Challenges for patients who could use the help from an NN, and were offered this help, are only reported in one earlier mentioned study [15]. This is not necessarily because none exist, but may be due to the methods used, where focus on the research has been on patients who were still offered access to a nurse navigator [12, 16]. Others have focused on benefits and not challenges [11, 13].

In the light of the disappointment the participants felt when offered an NN, it could be argued that instead of an NN, patients would be better helped boosting the healthcare system in general. Competences of an NN among others are to communicate very well. This was not what participants experienced when they were passed by the NN in the corridors without being greeted. In nursing culture a particular fast gait can be developed to signal being in a hurry and not wishing to be disturbed [38]. In our study such nonverbal signals were found especially inappropriate with regard to some patients, if omitted by an NN. This points to culturally embedded norms as challenges for healthcare professionals, if patients’ full satisfaction should be pursued. However, optimizing existing resources in the healthcare system might increase quality of healthcare in general, but if an NN is not offered for a period during a critical time, patients who could use this special kind of help could risk being left on their own.

### Limitations and strengths

A substantial group of consecutively included patients was followed before 11 patients were due to have surgery for cancer, and among these five patients could use the help. Due to the timeframe of this study only five patients provided data for this paper, showing an NN was an attachment figure when the help could be used. However, contrary to the five who could use the help, the six other patients who had surgery for cancer, and could not use the help, all had a known healthcare professional among their close relatives or a known primary physician from whom they felt sure to get help, if they asked (unpublished work). This supports the findings among the five that a healthcare professional attachment figure is of importance when women get cancer. Our sample originates from a single gynecological–obstetric department in Denmark, receiving patients from one region in Denmark (1.2 million inhabitants). The five participants presented diversities in age (37–76 years of age), marital status, place of residence and diagnostic phase when referred (Table 1). Albeit to different extents, they all received surgery as primary treatment. They had different gynecological diagnoses, and after treatment some were considered cured, others not. Some received chemotherapy as secondary treatment, but no participants received radiation therapy as secondary treatment. Moreover, some felt healthcare professionals to be very great authorities, others did not. Among these features no structure was found to support participants’ special experiences of an NN. Others have found female patients to have sexual problems after treatment for cancer in the reproductive system [39–41]. In this study no participant mentioned sexual problems in relation to help from the NN. On the contrary, the participants had good support by their partners or had not been sexually active for years. However, within the time frame of this study none of the participating women exceeded the limit of four weeks after surgery during which they were advised to refrain from sexual intercourse [42]. Furthermore, the NNs were female, and some sister solidarity could be expected, but nobody mentioned this. From our results no generalizations can be made, but rather a transfer to similar settings.

It should be noted that the NNs in this study had the time and urge to help the patients, and helped in areas not covered by others. Where others could help, they linked or took the contact to these helpers on behalf of the individual women. It cannot therefore be precluded that both personality and functions have been of importance for the result. On the contrary, the described combination created the offer of an emerging healthcare-related secure base, and must as such be seen as a whole.

A limited amount of data regarding experiences with the NN was available from one patient’s diaries, as she did not have the energy to continue to fill in the diary with contacts with the healthcare professionals after discharge. On the other hand, the longitudinal qualitative design with a) more ways to support participants to remember and b) the possibility of creating a relationship with the participants before the last interview was considered to contribute to participants being able and willing to share more experiences with the interviewer. Moreover, as the interviewer was not part of a healthcare team, participants felt free to talk about negative experiences in particular. This was a feeling they reported could not solely be provided by anonymity.

## Conclusion

Help from a nurse navigator (NN) was offered in an outpatient period before admission to a surgery ward, and from the patients’ point-of-view we found that those who could benefit from her help: a) collaboratively used help from all categories offered, including coordination, information and support, and requested help from the NN until one month after discharge; b) valued the single point of contact, the accessibility, the action taken to immediately help them, the time given and the professionalism, which included the NN’s knowledge and information style, her attention and support as well as the ability to reassure and empower patients; c) were especially seeking proximity to the NN and felt affectional bonds to her; and d) were disappointed due to feelings of rejection by the NN after her available period had passed (the bonds were broken). Attachment behavior; seeking proximity to a selected person with special competences is natural, and patients who could use the help from an NN expected the help to continue. NNs may risk provoking a break of affectional bonds, and making the patients’ trajectory of cancer “with seam instead of seamless”, if the NNs do not react as the patients expect: greet the patients, and help the patients. In the work of helping especially sick or scared patients to a better trajectory we therefore recommend an increased awareness of cultural areas in healthcare—like when to greet patients—as embedded norms might block for good communication. Moreover, we suggest a special awareness of critical periods in the patient’s trajectory, and for women we suggest the theory of attachment to supplement thoughts of continuity of care and coordination. In short, it is fine to offer additional help to those who can use it, but in practice as well as in research we draw attention to awareness on how and when to stop the help, if the patients are not going to be hurt. In practice, the first part of a subsequent less critical period could be used by the NN to prepare the patient and the primary physician (or a primary nurse) for a change in contact/attachment person by jointly working to obtain a secure relationship followed by a mutually agreed withdrawal.

## Reviewers

**Frede Olessen**, Professor, Research Unit for General Practice, Department of Public Health, University of Aarhus, Denmark

**Venke Sorlie**, PhD, RNT, Professor, Lovisenberg Diaconal University College, Oslo, Norway

One anonymous reviewer

## Figures and Tables

**Box 1.**
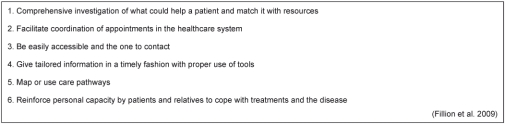
Key elements of the work of a nurse navigator

**Table 1.**
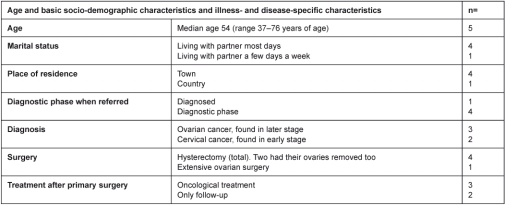
Distribution of participants on age and basic socio-demographic characteristics and illness- and disease-specific characteristics

**Table 2.**
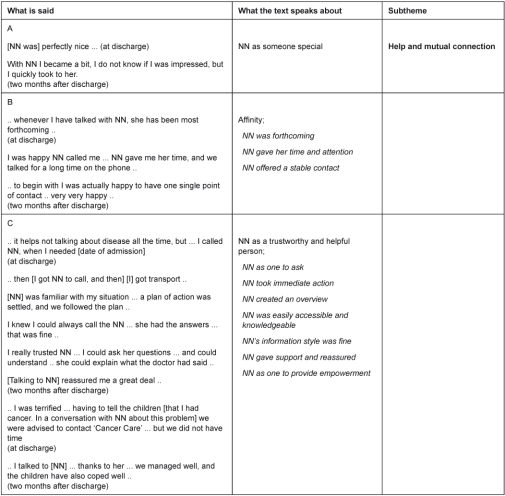
Theme: benefits for participants

**Table 3.**
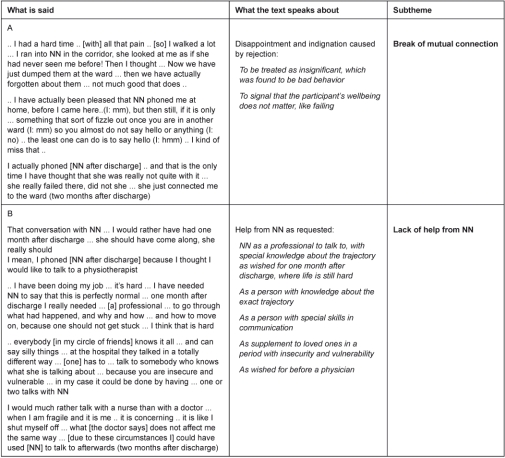
Theme: challenges for participants
